# Daily knowledge sharing at work: the role of daily knowledge sharing expectations, learning goal orientation and task interdependence

**DOI:** 10.1080/1359432X.2025.2458343

**Published:** 2025-01-30

**Authors:** Roy B. L. Sijbom, Ellis S. Emanuel, Jessie Koen, Matthijs Baas, Leander De Schutter

**Affiliations:** a School of Business and Economics, Department of Management and Organization, Vrije Universiteit Amsterdam, Amsterdam, The Netherlands; b Department of Work and Organizational Psychology, University of Amsterdam, Amsterdam, The Netherlands; c Department of Sustainable Productivity and Employability, Netherlands Organisation for Applied Scientific Research (TNO), Leiden, The Netherlands

**Keywords:** Daily knowledge sharing, daily knowledge sharing expectations, supervisor and co-workers, learning goal orientation, task interdependence

## Abstract

Knowledge sharing is vital for organizational success. Yet, most research treats it as a static behaviour, overlooking its fluctuations within individuals over time. Drawing on role theory and a cost-benefit framework, we argue that knowledge sharing expectations conveyed by supervisors and co-workers on a given day positively predict employees’ actual knowledge sharing on that day. Furthermore, we propose that learning goal orientation and task interdependence – key between-person characteristics – moderate this within-person relationship. We tested these hypotheses in two preregistered 10-day diary studies among UK employees (Study 1: 557 daily surveys from 101 respondents; Study 2: 401 daily surveys from 88 respondents). The results showed that daily knowledge sharing expectations are positively related to employees’ daily knowledge sharing, with the strongest effect size for co-worker knowledge sharing expectations. While perceived task interdependence did not moderate this day-level relationship, learning goal orientation showed varying moderating effects across studies: At higher levels of learning goal orientation, the positive day-level relationship was stronger in Study 1 but weaker in Study 2. Our study offers novel insights into the short-term nature of knowledge sharing and its boundary conditions, highlighting the importance of both daily knowledge sharing expectations and individual differences in shaping knowledge sharing in organizations.

Knowledge sharing, a discretionary behaviour involving the exchange of task-related information, advice, and expertise with others (Ipe, 2003; Wang & Noe, 2010), is crucial for employee learning and achieving work-related goals (Burmeister et al., 2021; Carmeli et al., 2013), as well as for organizational success (Argote & Ingram, 2000; Myers, 2021; Wang & Noe, 2010). Despite the benefits of knowledge sharing, individuals within organizations are not always inclined to do so. For example, individuals may be ignorant that colleagues could benefit from their knowledge for everyday work-related challenges, may feel that knowledge sharing is not part of their everyday job, or they may hesitate to share valuable knowledge because doing so could weaken their competitive position among peers (Ardichvili et al., 2003; Israilidis et al., 2021).

In the search for factors that predict knowledge sharing, existing research has predominantly focused on knowledge sharing as a static and stable construct and examined its relationship with other static and stable variables, such as personality (Chiaburu et al., 2011; Matzler & Mueller, 2011), leadership styles (Srivastava et al., 2006) and job design characteristics (Foss et al., 2009). However, recent studies highlight that knowledge sharing also exhibits substantial short-term, within-person variability, with fluctuations from day to day accounting for approximately half of the variance (Li et al., 2022). This dynamic perspective is crucial because understanding how short-term changes in these behaviours occur could inform theory building on knowledge sharing in action, as emphasized in prior within-person research on knowledge exchange behaviours (e.g., Venz & Mohr, 2023; Venz & Nesher Shoshan, 2022). Moreover, such an understanding can help organizations promote incremental improvements in knowledge sharing behaviours on a daily basis, which may yield significant cumulative benefits over time.

Fluctuations in knowledge sharing may arise because on some days employees feel more encouraged by others to share knowledge than on other days, which we refer to here as *knowledge sharing expectations*. In workplace settings, such expectations typically originate from supervisors and co-workers because they most frequently interact with employees (Raveendran et al., 2020). However, expectations and encouragements from others within the organization are often not documented or supported by formal systems and easily dissipate without reinforcement (Israilidis et al., 2021). For knowledge sharing expectations to remain salient, regular reinforcement through explicit encouragement is needed. Despite this, little research has directly investigated these expectations, particularly how and when their daily affirmation might predict daily knowledge sharing.

Our study addresses two key questions. First, how do daily knowledge sharing expectations predict the daily knowledge sharing of employees? Drawing on role perception theory (Dierdorff & Morgeson, 2007) and a cost-benefit framework (cf. Morrison & Vancouver, 2000; Wang & Noe, 2010), we argue that daily expressions of knowledge sharing expectations signal to employees that knowledge sharing is a core job responsibility, lowering perceived costs and enhancing perceived benefits of knowledge sharing behaviours, thereby enhancing knowledge sharing. Second, does this relationship depend on personal and job characteristics? We propose that learning goal orientation (a personal trait) and task interdependence (a job characteristic) influence the extent to which employees naturally view knowledge sharing as an integral responsibility within their roles. Individuals who score low on these characteristics may need explicit prompts or expectations to engage in daily knowledge sharing. Learning goal orientation reflects an intrinsic desire to enhance abilities by acquiring new skills, mastering unfamiliar situations, and improving competence (VandeWalle, 1997). Because individuals with high levels of learning goal orientation are motivated to share knowledge for intrinsic reasons (e.g., learning and development; Matzler & Mueller, 2011; Shariq et al., 2019), they may be less influenced by external cues like daily knowledge sharing expectations. Similarly, under conditions of task interdependence, defined as the need for team members to exchange resources to perform their jobs (Van der Vegt & Van de Vliert, 2002), knowledge sharing is naturally higher, diminishing the dependency on daily expectations. Thus, we hypothesize that both learning goal orientation and task interdependence are associated with a reduced reliance on daily knowledge sharing expectations, thus weakening the positive relationship between these expectations and knowledge sharing. We examine our ideas in two preregistered daily diary studies conducted over 10 workdays. Our conceptual model is shown in Figure 1.

**Figure 1.**
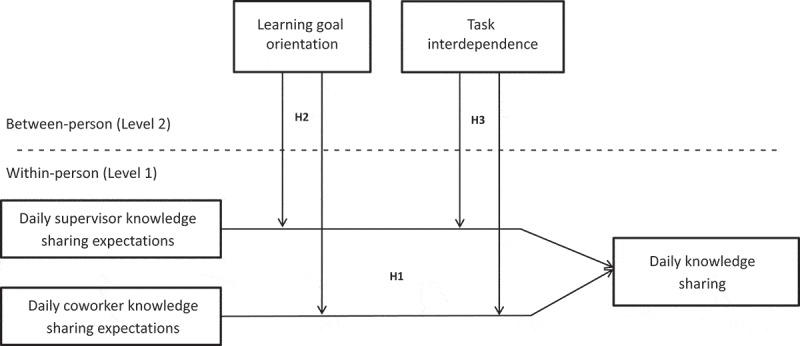
Research model.

This study makes two important contributions to the literature. First, by investigating the relationship of daily knowledge sharing expectations and knowledge sharing, we respond to recent calls for research on the daily antecedents of knowledge sharing (Ford et al., 2015; Li et al., 2022; Zhang & Jiang, 2015). Given that knowledge sharing shows substantial within-person variation, investigating knowledge sharing expectations will contribute to building a nomological network of knowledge sharing at the within-person level (Dalal et al., 2009). Moreover, this study highlights the practical importance of reinforcing daily knowledge sharing expectations to encourage knowledge exchange. Second, by investigating learning goal orientation and task interdependence as between-person boundary conditions, we identify whose (i.e., individuals with lower levels of learning goal orientation and individuals with lower levels of task interdependence) knowledge sharing is particularly sensitive to daily fluctuations in knowledge sharing expectations. Thus, our study expands the understanding of short-term knowledge sharing interactions at work.

## Theoretical framework

### The daily occurrence of knowledge sharing in organizational contexts

Knowledge sharing is voluntary in nature (Gagne, 2009) and organizations depend on employees’ willingness and ability to share their knowledge with colleagues on a daily basis (Afshar Jalili & Ghaleh, 2021). The cost-benefit framework (cf. Morrison & Vancouver, 2000; Wang & Noe, 2010) provides a valuable lens for understanding within-person variations in knowledge sharing. Sharing knowledge can be costly because, once disclosed, the previously private knowledge may lose its value or competitive advantage (Park et al., 2017). However, sharing knowledge also offers potential benefits such as increased recognition, enhanced status, or returned favours. Therefore, in deciding whether or not to disclose knowledge in everyday work interactions, employees weigh potential benefits against the potential costs (Cabrera et al., 2006; Nonaka, 1994; Wang & Noe, 2010). This implies that knowledge sharing can be enhanced if potential benefits are increased or potential costs are reduced (Cabrera & Cabrera, 2002). Knowledge sharing expectations might be relevant in this regard, as we explain below.

### Knowledge sharing expectations

According to role theory (Dierdorff & Morgeson, 2007), role expectations emerge from a “role-set”, typically supervisors and co-workers (Biddle, 1986), with whom employees interact. Employees gain clarity about appropriate behaviours through communication with their supervisors and co-workers (Katz & Kahn, 1978; Raveendran et al., 2020), which is particularly crucial in ambiguous work roles where formal and informal expectations may be unclear (Ilgen & Hollenbeck, 1991), as is the case with knowledge sharing. Such communication not only clarifies expectations regarding knowledge sharing but also help employees understand the potential rewards (e.g., social reinforcement, recognition) and costs (e.g., losing competitive advantage, not meeting expectations when someone does not share knowledge) associated with showing knowledge sharing behaviours (Biddle, 1986; Katz & Kahn, 1978). By highlighting behaviours that are crucial for performance and social approval, role expectations become crucial for effective role enactment. Indeed, previous research has shown positive associations between specific role expectations and corresponding work outcomes, such as the association between creativity expectations and employee creativity (Qu et al., 2015) and between voice expectations and employee voice (Kakkar et al., 2016). In a similar vein, we expect positive associations between knowledge sharing expectations and knowledge sharing, with knowledge sharing expectations referring to beliefs or assumptions about the extent to which individuals in a particular role are expected to share knowledge, information, or expertise with others.

Although previous research often conceptualizes role expectations as a stable factor that describes what is generally expected from individuals by their supervisors and co-workers, there are compelling reasons to believe that knowledge sharing expectations fluctuate daily. First, knowledge sharing is typically informal in nature and not explicitly outlined in role descriptions, creating ambiguity around whether, when, and how much employees are expected to share their knowledge. Daily interactions with supervisors or colleagues may heighten, reduce, or leave unaddressed these expectations, depending on the specific requests or (lack of) encouragements that are made (Israilidis et al., 2021). Second, employees may not interact with their supervisors or co-workers every day, leading to variability in the salience of knowledge sharing expectations. These expectations, reinforced through direct communication and collaboration, can shift based on whether employees engage with others on a given day. Taken together, in the current research, we adopt a dynamic perspective and focus on daily knowledge sharing expectations. In the following, we describe how and when daily fluctuations in knowledge sharing expectations predict employee daily knowledge sharing.

### Daily knowledge sharing expectations as a predictor of daily knowledge sharing

Drawing on role theory (Dierdorff & Morgeson, 2007), we argue that daily knowledge sharing expectations play a crucial role in shaping employees’ daily knowledge sharing, as they help reduce perceived costs and enhance the perceived benefits of sharing knowledge. Since knowledge sharing is often not formalized in job roles, these daily expectations provide clarity on whether to enact knowledge sharing on a daily basis (cf. Jackson, 1981). That is, these expectations signal to employees “what is important and what behaviors are expected and rewarded” (Ostroff & Bowen, 2016, p. 204), making employees more likely to engage in knowledge sharing when it is perceived as an appreciated part of their role.

Knowledge sharing expectations, often communicated by supervisors and co-workers (Raveendran et al., 2020), serve as reminders that clarify and reinforce the importance of knowledge sharing. These reminders help the role occupant understand that sharing of knowledge is an expected and appreciated part of their role. This aligns with the theory of planned behaviour (Ajzen, 1991), which suggests that individuals are more likely to engage in behaviours they perceive as socially approved. When employees anticipate positive social outcomes, such as appreciation for sharing knowledge, they are more motivated to engage in knowledge sharing. In addition, knowing that knowledge sharing is expected within the organization reduces the tendency for employees to view knowledge as a personal asset to keep to themselves (Israilidis et al., 2021). In fact, they may be reluctant to withhold knowledge to avoid social disapproval. Hence, daily reminders encourage employees to share information by reducing perceived risks, such as disapproval, disappointment, or loss of competitive advantage, while increasing the likelihood of receiving social rewards, such as recognition and appreciation. Moreover, knowledge sharing expectations clarify the utility of sharing knowledge. Employees may not always realize that their colleagues can benefit from their knowledge in their daily work (Israilidis et al., 2021). Given their direct and explicit appeal, we expect knowledge sharing expectations and requests will predict immediate knowledge sharing behaviours. This logic is supported by a study conducted by Appel-Meulenbroek et al. (2018) which showed that when a co-worker requests information during unplanned work-related meetings, employees are likely to share the requested information promptly.

From a cost-benefit perspective, daily knowledge sharing expectations clarify role expectations, enhance anticipated appreciation, and reduce anticipated risks, thereby positively predicting same-day knowledge sharing. Initial evidence for this proposition comes from a study showing that daily managerial coaching, which includes clarifying performance expectations, is positively related to knowledge sharing with colleagues the same day (Li et al., 2022). Additionally, Xia et al. (2022) showed that polite requests for knowledge sharing were indirectly negatively related to knowledge hiding the same day. By making knowledge sharing expectations clear, employees feel more supported and are more likely to take a positive attitude towards sharing, further reducing perceived risks (Bashir & Sang Long, 2015; Chiaburu & Harrison, 2008). Thus, we hypothesize:

Hypothesis 1: Daily perceived knowledge sharing expectations are positively related to daily knowledge sharing.

We do not propose specific hypotheses about differences in the strength of expectations from supervisors versus co-workers as the relative influence of these sources remains unclear. Supervisors may exert stronger influence on role expectations due to their power to control resources and enforce rules (Hogg & Tindale, 2008; Pfeffer, 1993). In contrast, co-workers, through frequent daily interactions, may be more effective in encouraging knowledge sharing behaviours due to stronger social bonds and peer influence (Dannals et al., 2020; Siemsen et al., 2009). While both supervisors and co-workers are primary sources that shape norms and role expectations in the workplace (Raveendran et al., 2020), it remains unclear whether their influence differs in strength. As such, we hypothesize direct relationships for both. However, we will exploratory examine potential differences in the strength of their influence.

### The moderating role of between-person characteristics

We argue that daily knowledge sharing expectation cues are positively related to daily knowledge sharing. However, employees may not respond uniformly to these cues. Drawing on the idea that personality traits and workplace characteristics shape individual responses to their work environment (e.g., van Knippenberg & Hirst, 2020; Wang & Noe, 2010), we examine learning goal orientation and task interdependence as between-person moderators. From a substitute perspective (Kerr & Jermier, 1978), which posits that certain individual or situational factors can reduce or negate the impact of external leadership or environmental cues, we argue that employees with higher levels of learning goal orientation or those working on more (vs. less) interdependent tasks may consider knowledge sharing as more (vs. less) integral to their work role. As such, these employees are less reliant on daily cues from others to engage in knowledge sharing. Therefore, these between-person factors may diminish the extent to which daily knowledge sharing expectations predict knowledge sharing behaviours.

#### Learning goal orientation as a between-person moderator

Knowledge sharing is a deliberate and conscious behaviour where individuals to a considerable extent choose to exchange information based on their internal dispositions and motivation (Ipe, 2003). A key dispositional motivational factor in this regard is learning goal orientation, defined as a desire to develop skills, master new situations, and enhance personal competence (VandeWalle, 1997). Employees with high learning goal orientation are intrinsically motivated and seek learning opportunities (Janssen & Van Yperen, 2004).

Individuals with a high learning goal orientation are likely to engage in knowledge sharing behaviours naturally, relying less on external triggers such as supervisors’ and co-workers’ knowledge sharing expectations. For these individuals, knowledge sharing is intrinsically motivating because it provides a means for personal growth and contributes to collective development (Matzler & Mueller, 2011; Shariq et al., 2019). As a result, they are less driven by extrinsic cost-benefit analyses (e.g., maintaining a competitive edge by withholding valuable information; Lu et al., 2012; Poortvliet et al., 2007). Moreover, individuals high in learning goal orientation perceive knowledge sharing as an intricate part of their role and identity, requiring no external prompt or encouragement. Supporting this, Chadwick and Raver (2015) found that high levels of learning goal orientation are associated with the adoption of exploratory strategies, which make employees more proactive in seeking out learning opportunities and less reliant on others for guidance. Additionally, employees with a high learning goal orientation engage in behaviours that facilitate interaction and knowledge exchange, such as seeking feedback (Janssen & Prins, 2007), supporting colleagues (Porter, 2005), and discussing work-related issues (Gray & Meister, 2004). As such, for individuals with high learning goal orientation, daily knowledge sharing is less dependent on external situational cues, such as daily expectations from supervisors and co-workers to share knowledge. In contrast, individuals with low learning goal orientation may have a reduced intrinsic drive to share knowledge and instead may rely more on regular prompts from others in their environment to do so.

Therefore, we propose that learning goal orientation moderates the within-person relationship between knowledge sharing expectations and knowledge sharing behaviour. Specifically, this within-person relationship will be stronger for employees with lower levels of learning goal orientation, who depend more on external expectations, and weaker for employees with higher learning goal orientation, who share knowledge for intrinsic reasons. In this way, high learning goal orientation can be viewed as a substitute for daily knowledge sharing expectations.

Hypothesis 2: Employees’ learning goal orientation moderates the positive within-person relationship between knowledge sharing expectations and knowledge sharing such that this within-person relationship will be stronger (weaker) for employees with a lower (higher) level of learning goal orientation.

#### Task interdependence as a between-person moderator

Task interdependence refers to the extent to which employees must share materials, information, or expertise to achieve desired performance outcomes (Van der Vegt & Van de Vliert, 2002). When task interdependence is high, individuals rely on each other to perform tasks, which necessitates the coordination of effort, actions, resources, and expertise among members. Indeed, sharing resources, including information, is vital for highly interdependent tasks (Brass, 1981; Staples & Webster, 2008). Moreover, for highly interdependent tasks, employees naturally discuss the (coordination of) roles, tasks and deliverables with each other, thereby resulting in perceptions of knowledge sharing being an intricate part of their interdependent roles. Also, due to their interdependence, employees may be less concerned about losing competitive positions and have less reason to withhold valuable information. Thus, given that high task interdependence already involves significant knowledge sharing, employees’ daily knowledge sharing is assumed to be less dependent on daily knowledge sharing expectations, thereby fulfilling a (partly) substituting function.

In contrast, when task interdependence is low, employees’ work does not inherently require knowledge sharing, as tasks can be completed independently. In such settings, work design requirements do not imply that knowledge sharing is part of their role (Fong et al., 2018; Staples & Webster, 2008). Under these conditions, explicit prompts for daily knowledge sharing may become more important by providing motivational cues and clarifying role expectations regarding knowledge sharing. Therefore, we expect the positive within-person relationship between daily knowledge sharing expectations and knowledge sharing to be stronger when task interdependence is low rather than high. Accordingly, we hypothesize:

Hypothesis 3: *Employees’ task interdependence moderates the positive within-person relationship between knowledge sharing expectations and knowledge sharing such that this within-person relationship will be stronger (weaker) for employees who have a lower (higher) level of task interdependence.*

## Study 1: Method

### Transparency and openness

We described our sampling plan, all data exclusions, and all measures used in the study. All data, analysis code, and research materials are available on the OSF page. This study’s design and analysis were preregistered (https://osf.io/un7k5).

### Procedure

We used an experience sampling method (ESM) to collect diary data from participants. This approach captured not only a snapshot of employees’ experiences during a specific period but also how these experiences fluctuated over time (Beal, 2015; Ohly et al., 2010). The key variables in our study—supervisor and co-worker knowledge sharing expectations, as well as knowledge sharing itself— were expected to vary from day to day. Following recommendations by Fisher and To (2012), we collected diary data on consecutive days rather than weekly.

Relying on the rule of thumb to sample at least 100 employees (Ohly et al., 2010) and have a Level-2 sample size of at least 83 (Gabriel et al., 2019), we oversampled to manage potential dropout. We recruited 120 participants via the online recruitment platform Prolific Academic, which produces data of good quality (Douglas et al., 2023). To be eligible to participate in our study, participants had to be between 18 and 67 years old, speak English, work at least 31 hours per week, report to a direct supervisor, and regularly interact with other employees (e.g., co-workers, colleagues, subordinates). The data collection occurred in two phases. First, we measured participants’ demographics, trait learning goal orientation, and task interdependence. Second, one week after the baseline survey, participants completed a daily survey for 10 consecutive workdays. The daily survey measured participants’ perceptions of knowledge sharing expectations from their supervisors and co-workers, and their self-reported knowledge sharing. The study was approved by the faculty research ethics board (#2022-WOP-15320) and conducted between 29 June and 15 July, 2022.

Participants provided informed consent before completing the baseline survey, for which they received £1.35 (duration: approximately 3 minutes and 45 seconds). The daily surveys were sent at the end of each workday (5:30 pm), with reminders at 8:30 pm and a completion deadline of midnight. Each completed survey earned participants £0.67 and took approximately one minute to complete. To properly understand the relationship between daily knowledge sharing expectations and daily knowledge sharing, we verified each day whether participants interacted with their supervisor and/or co-workers. Questions regarding supervisor knowledge sharing expectations and co-worker knowledge sharing expectations were presented if participants indicated they interacted with them on that day. If no interactions occurred, participants only reported their knowledge sharing for that day to prevent participants completing daily surveys without answering any questions. This protocol ensured consistent and comparable survey responses.

### Sample

The initial sample included 120 employed workers from the United Kingdom who completed the baseline survey, and resulted in 949 daily observations. We excluded five participants who only completed the baseline survey. Additionally, we removed 118 daily observations where participants reported no interactions with their supervisor or co-workers. Finally, to enable a minimum of within-person variation, we included only participants who completed both the baseline survey and daily survey on at least two workdays (Nezlek, 2011), leading to the exclusion of four participants. Among the remaining 827 daily surveys, 641 included days where an employee interacted with their supervisor only, 750 included days with only co-worker interactions, and 564 included days where employees interacted with both their supervisor and a co-worker. Here we take the sample that included interactions with both supervisors and co-workers for at least two days, excluding an additional seven participants, leading to a final sample of 557 days and 101 participants. On average, participants completed 5.51 daily surveys (*SD* = 2.52).

The sample included 42 men (41.60%) and 57 women (56.40%), with two participants (2.00%) opting not to disclose their gender. The average age was 36.38 years (*SD* = 9.31), and the mean organizational tenure at the current workplace was 7.48 years (*SD* = 7.33). Most participants worked 31–40 hours per week (78.10%), while others worked 41–50 hours (20.80%) or 51–60 hours (1.10%). Nearly all participants (99.00%) held at least a high-school degree, and 72.30% had a college or university degree.

### Measures

All variables were assessed using a 5-point Likert-type response scales ranging from 1 (*strongly disagree*) to 5 (*strongly agree*). Table 1 displays the scales’ reliabilities, means, standard deviations (SDs), intraclass correlations (ICCs), and within-person and between-person correlations among the study variables.

**Table 1.** Descriptive statistics, Cronbach’s alphas, intraclass correlation coefficients, and correlations between study variables (study 1).

Variable		*M*	*SD*_b_	*SD*_w_	α_b_	α_w_	ICC	1	2	3	4	5
1	Daily supervisor knowledge sharing expectations	3.88	0.60	0.82	.90	.86	.35		.58**	.51**	.24*	.19
2	Daily co-worker knowledge sharing expectations	3.95	0.55	0.70	.88	.85	.45	.48**		.81**	.37**	.30**
3	Daily knowledge sharing	4.16	0.45	0.65	.94	.92	.30	.44**	.71**		.36**	.21**
4	Learning goal orientation	4.10	0.66		.90							.13
5	Task interdependence	3.96	0.56		.69							

Note: Means, standard deviations at the between-person level (SD_b_) and within-person (i.e., day) level (SD_w_), and within-person correlations (*N* = 577; below diagonal) and between-person correlations (*N* = 101; above diagonal) among study variables are displayed. Cronbach’s alphas for the between-person level (α_b_) and for the within-person level (α_w_) are depicted. ICC = intraclass correlation.

*p < .05; **p < .001.

#### Baseline survey measures

##### Learning goal orientation

We measured learning goal orientation using a five-item scale from VandeWalle (1997). A sample item is: “I often look for opportunities to develop new skills and knowledge”.

##### Task interdependence

We measured task interdependence with five items from Van der Vegt et al. (2000). A sample item is: “I need information and advice from my colleagues to perform my job well”. One negatively phrased item was recoded (“For my job, it is not necessary for me to coordinate or cooperate with others”). Cronbach’s alpha for this scale was .56, but after removing the negatively phrased item, it improved to .69. Therefore, we used the four-item scale in further analyses.

#### Daily survey measures

##### Perceived supervisor [co-worker] knowledge sharing expectations

We assessed daily perceived supervisor [co-worker] knowledge sharing expectations using three items adapted from the Peer Support subscale of the Learning Transfer System Inventory (LTSI) by Holton et al. (2000). Items were: “Today, my supervisor [co-workers] encouraged me to share knowledge”, “Today, my supervisor [co-workers] appreciated me sharing knowledge”, and “Today, my supervisor [co-workers] expected me to share knowledge”.

##### Knowledge sharing

We measured the extent to which participants shared knowledge using items adapted to the daily level from Hsu et al. (2007), which were initially developed by Davenport and Prusak (1998). The scale from Hsu and colleagues (2007) was designed to measure knowledge sharing in an online community, which differs from our research context. Therefore, we adapted one of the items about employees’ willingness to share knowledge and removed redundant information (e.g., two items about online topics and discussions). This led to the following four items: “Today, I actively participated in knowledge sharing with my colleagues”, “Today, I took time to share knowledge with my colleagues”, “Today, I actively shared knowledge with my colleagues”, and “Today, I was willing to share my knowledge with my colleagues”.

### Measurement model

To evaluate the measurement model, we conducted two types of confirmatory factor analyses. First, we performed a multilevel confirmatory factor analysis (MLCFA) using Mplus 8.0 (Muthén & Muthén, 1998-2017) to test the study variables’ convergent and discriminant validity. We compared a three-factor model (daily knowledge sharing, daily supervisor knowledge sharing expectations, and daily co-worker knowledge sharing expectations), which we specified at both the day level (within-level) and the person level (between-level) using MLR, with alternative models. The three-factor model showed a good fit (*χ*^2^(64) = 167.994, *p* < .001, comparative fit index (CFI) = .956, Tucker-Lewis Index (TLI) = .939, root-mean-square error of approximation (RMSEA) = .054, standardized root mean square residual (SRMR)_within_ = .037, SRMR_between_ = .073), and fit the data significantly better than a two-factor model, where supervisor and co-worker knowledge sharing expectations items loaded onto the same factor, (*χ*^2^(68) = 684.297, *p* < .001, CFI = .741, TLI = .657, RMSEA = .128, SRMR_within_ = .117, SRMR_between_ = .182), and a one-factor model (*χ*^2^(70) = 1453.565, *p* < .001, CFI = .418, TLI = .252, RMSEA = .188, SRMR_within_ = .142, SRMR_between_ = .172). Second, we conducted a confirmatory factor analysis (CFA) with the person-level variables. A two-factor model (learning goal orientation and task interdependence) showed a good fit, *χ*^2^(26) = 38.475, *p* = .055, CFI = .961, TLI = .946, RMSEA = .029, SRMR = .054), and was significantly better than a one-factor model (*χ*^2^(27) = 95.209, *p* < .001, CFI = .788, TLI = .718, RMSEA = .067, SRMR = .140).

#### Analytical strategy

Our data had a hierarchical structure, with daily assessments (Level 1) nested within employees (Level 2). To address this nested structure, we employed multilevel modelling using Mplus 8.0 (Muthén & Muthén, 1998-2017). Following recommendations in the literature (Aguinis et al., 2013; Hox et al., 2017; Ohly et al., 2010), we conducted our analyses in a four-step procedure. In Step 1, we estimated the within-person and between-person variability in knowledge sharing by estimating the intra-class correlation type 1 (ICC) on the basis of a null model with only a random intercept. The ICC indicates how much variance of knowledge sharing is explained at the between-person level.

In Step 2, we added the predictor variables to the model to examine their main effects on daily knowledge sharing. We included time (the 10 daily study occasions) as a Level-1 control variable. Following standard recommendations (Gabriel et al., 2019; Hofmann et al., 2000), we person-mean centred Level-1 (day-level) predictors (daily supervisor knowledge sharing expectations and daily co-worker knowledge sharing expectations), and grand-mean centred Level-2 (person-level) predictors (learning goal orientation and task interdependence). Step 2 already allowed us to test Hypothesis 1 regarding main effects. In addition, we conducted exploratory analyses to determine whether the coefficients for supervisor and co-worker knowledge sharing expectations (from the joint model) differ from each other.

In Step 3, we examined random slope variability in the two Level-1 predictors by adding random effects for these two predictors and covariance between these random effects to the models. Random slope variability in Level-1 predictors is of interest for interpreting the magnitude of possible subsequent findings regarding cross-level interaction effects as it suggests that the effect of the Level-1 predictors might vary across individuals.

In Step 4, we modelled the cross-level interaction terms in Mplus by specifying the slope of the Level-1 predictor as a random coefficient that varies across Level-2 units (i.e., individuals). At Level 2, we regressed this random slope on the moderator variables. A significant result would indicate that some of the variance in the random slope of the Level-1 variable is explained by differences in the Level-2 variable. When the Level-2 variable significantly predicted variance in the random slope of the Level-1 predictor, we further investigated the nature of this moderating effect by calculating the conditional effects of the Level-1 predictor at different levels of the moderator (mean ±1 SD) using the MODEL CONSTRAINT function in Mplus 8.0 (Muthén & Muthén, 1998-2017; Preacher et al., 2006).

## Study 1: results

Table 2 presents the results of the multilevel regression models. Results from Step 1 showed an ICC estimate of .30, indicating that 30% of the variance in knowledge sharing is between person and 70% is within person. In line with Hypothesis 1, daily perceived supervisor and co-worker knowledge sharing expectations were positively related to daily knowledge sharing, estimate = 0.11, *SE* = 0.03, *p* < .001 and estimate = 0.59, *SE* = 0.06, *p* < .001, respectively (see Step 2). Results from an exploratory test showed that the difference in strength between these two estimates was significant (difference = −0.48; *SE* = 0.08; *p* < .001), indicating that co-worker knowledge sharing expectations had a significantly stronger relationship with knowledge sharing than supervisor knowledge sharing expectations.

**Table 2.** Results of multilevel modelling analysis (study 1).

		*Model*					
		*Null (Step 1)*	*Random Intercept and Fixed Slope (Step 2)*	*Random Intercept and Random Slope (Step 3)*	*Cross-level interaction (Step 4a)*	*Cross-level interaction (Step 4b)*	*Cross-level interaction (Step 4)*
		*b (SE)*	*b* (SE)	*b* (SE)	*b* (SE)	*b* (SE)	*b* (SE)
*Fixed effects parameters*							
	*Within-person level*						
	Intercept	4.13*** (0.04)	4.16*** (0.06)	4.15*** (0.05)	4.15*** (0.05)	4.16*** (0.05)	4.15*** (0.05)
	Day		−0.00 (0.01)	−0.00 (0.01)	−0.00 (0.01)	−0.00 (0.01)	−0.00 (0.01)
	Daily supervisor knowledge sharing expectations (DSKSE)		0.11*** (0.03)	0.11*** (0.03)	0.12*** (0.03)	0.11*** (0.03)	0.12*** (0.03)
	Daily co-worker knowledge sharing expectations (DCKSE)		0.59*** (0.06)	0.49*** (0.06)	0.49*** (0.06)	0.49*** (0.06)	0.49*** (0.06)
	*Between-person level*						
	Learning goal orientation (LGO)		0.23*** (0.06)	0.27*** (0.06)	0.23*** (0.06)	0.27*** (0.06)	0.23*** (0.06)
	Task interdependence (TI)		0.13 (0.07)	0.15* (0.07)	0.15** (0.07)	0.13 (0.07)	0.14 (0.07)
	*Cross-level interactions*						
	DSKSE × LGO				−0.08 (0.06)		−0.08 (0.06)
	DCKSE × LGO				0.30** (0.11)		0.29** (0.11)
	DSKSE × TI					−0.03 (0.05)	−0.02 (0.05)
	DCKSE × TI					0.14 (0.11)	0.12 (0.12)
*Random effects parameters*							
	σ^2^ within	0.30*** (0.04)	0.18*** (0.02)	0.15* (0.02)	0.15*** (0.03)	0.15*** (0.02)	0.15*** (0.03)
	σ^2^ between	0.13*** (0.03)	0.13*** (0.02)	0.14*** (0.02)	0.14*** (0.02)	0.14*** (0.02)	0.14*** (0.02)
	σ^2^ slope DSKSE			0.01 (0.04)	0.01 (0.04)	0.01 (0.04)	0.01 (0.04)
	σ^2^ slope DCKSE			0.15** (0.06)	0.13** (0.05)	0.14** (0.05)	0.12* (0.05)
	ICC	0.30					
	Deviance (MLR)	511.94	391.87	372.49	367.61	371.87	367.12
	*R*^2^ (Level 1)		0.389	0.503	0.510	0.504	0.510
	*R*^2^ (Level 2)		0.023	−0.054	−0.054	−0.054	−0.054

MLR = maximum likelihood estimation with robust standard errors. Fixed effects represent the average relationships between the predictors and the outcomes across all individuals. Random effect parameters capture the variance in daily knowledge sharing at both the within- and between-person levels, as well as the variance in the slopes of daily knowledge-sharing expectations that is not explained by Level 2 predictors.

*p < .05. **p < .01. ***p < .001.

**Figure 2.**
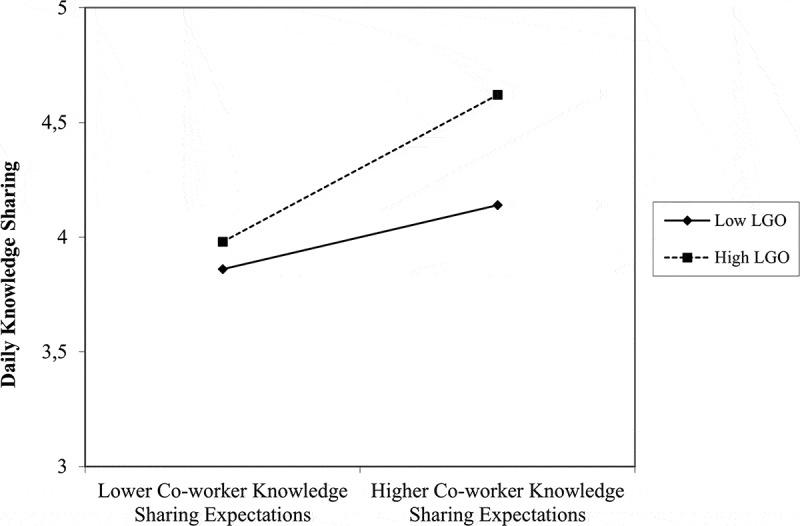
Dispositional learning goal orientation (LGO) as a cross-level moderator of the day-specific relationship between co-worker knowledge sharing expectations and knowledge sharing (study 1).

We hypothesized that learning goal orientation would moderate the positive relationship between daily perceived knowledge sharing expectations by supervisors and co-workers and day-level knowledge sharing, such that the relationship would be weaker for employees with higher rather than lower levels of learning goal orientation. Learning goal orientation did not moderate the effect of daily supervisor knowledge sharing expectations (estimate = −0.08, *SE* = 0.06, *p* = .170), but did moderate the effect of daily co-worker knowledge sharing expectations on knowledge sharing (estimate = 0.29, *SE* = 0.11, *p* = .006; see Step 4 of Table 2). Simple slope analysis showed that the positive relation between daily co-worker knowledge sharing expectations and daily knowledge sharing is weaker at lower levels (−1 SD) of learning goal orientation (estimate = 0.30, *SE* = 0.11, *p* = .005) than at higher levels (+1 SD) of learning goal orientation (estimate = 0.68, *SE* = 0.08, *p* < .001). This interaction effect is plotted in Figure 2. In all, these results did not support Hypothesis 2 because the interaction was either not significant (for supervisors) or the pattern was inconsistent with our hypothesis (for co-workers).

We hypothesized that task interdependence would moderate the positive relation between daily perceived knowledge sharing expectations and daily knowledge sharing. Results showed no significant interaction effect of task interdependence with daily knowledge sharing expectations by supervisors (estimate = −0.02, *SE* = 0.05, *p* = .646) or co-workers (estimate = 0.12, *SE* = 0.12, *p* = .334; see Step 4 of Table 2). Thus, Hypothesis 3 was not supported.

### Supplementary analyses

To complement the main analyses, we conducted supplementary analyses to examine the effects of daily supervisor and co-worker knowledge sharing expectations in isolation – so without controlling for the other source of knowledge sharing expectations (available as Tables S1.1 and S1.2 in supplementary material). These analyses showed that the positive relationship between daily perceived supervisor knowledge sharing expectations and knowledge sharing remained significant (estimate = 0.28, *SE* = 0.05, *p* < .001; Step 2 Table S1.1), as well as the relationship between co-worker knowledge sharing expectations and knowledge sharing (estimate = 0.67, *SE* = 0.05, *p* < .001; Step 2 Table S1.2). None of the interaction effects involving learning goal orientation and task interdependence were significant (see Step 4 of Table S1.1 and Table S1.2). Thus, the significance of the interaction effect of co-worker knowledge sharing expectations and learning goal orientation depends on the inclusion of supervisor knowledge sharing expectations as a covariate.

We further examined whether knowledge sharing and knowledge sharing expectations from the previous day predict knowledge sharing on the subsequent day. The correlations are reported in Table S1.3. Knowledge sharing was negatively correlated with knowledge sharing the following day (*r* = −.17, *p* = .002). Further, neither supervisor daily knowledge sharing expectations (*r* = −.08, *p* = .126), nor co-worker daily knowledge sharing expectations (*r* = −.08, *p* = .153) were significantly related to knowledge sharing on the following day.

### Discussion study 1 and introduction to study 2

The findings from Study 1 confirm the predicted direct relationship between daily knowledge-sharing expectations from both supervisors and co-workers and daily knowledge sharing by employees. This relationship was stronger when such expectations came from co-workers. Our hypotheses about cross-level moderators, however, were not supported. Specifically, task interdependence did not influence the daily relationship between expectations and knowledge sharing behaviour and learning goal orientation showed an unexpected pattern: While we anticipated that learning goal orientation would weaken the relationship between expectations and knowledge sharing behaviour, we found that employees with high learning goal orientation were more, rather than less, likely to share knowledge in response to daily co-worker expectations. Note that this interaction effect was significant only when supervisor knowledge-sharing expectations were included as a covariate.

To ensure the robustness of Study 1’s findings, we conducted a replication study (Study 2) using a similar research design. This second study incorporated additional attention checks to enhance confidence in data quality and we included alternative factors that may explain fluctuations in daily knowledge sharing. First, we included daily negative affect as a within-person variable control variable to rule out negative affect as a potential alternative explanation for our findings. Negative affect, associated with resource depletion (Bruyneel et al., 2009) and goal inhibition (Moberly & Watkins, 2010), can influence knowledge sharing behaviours, which require intentional effort and are often critical for achieving work-related goals. Furthermore, knowledge-sharing decisions involve perceived risks, such as losing a competitive advantage or facing social disapproval, both of which have been linked to negative affect (Sobkow et al., 2016). By controlling for negative affect, we aim to strengthen the validity of our conclusions.

Second, we examined whether daily general support might act as an additional – or potentially more influential – predictor of knowledge-sharing behaviour. Employees may share knowledge not only in response to explicit expectations from supervisors and co-workers but also as a way to reciprocate general supportive behaviours they experience (Myers, 2021; Obrenovic et al., 2020). If daily general support explains a larger proportion of variance in knowledge sharing behaviour, this would align with the social exchange perspective (Cropanzano et al., 2017), emphasizing reciprocal actions over role theory and the cost-benefit framework (i.e., that employees share knowledge in response to communicated expectations that clarify and reinforce the importance of knowledge sharing in their work roles). Conversely, if daily knowledge sharing expectations retain incremental validity even when daily general support is accounted for, this would reinforce the robustness and theoretical value of our findings.

Third, we considered reciprocity norms as another alternative explanation for knowledge-sharing behaviour: employees may habitually share knowledge due to established norms of reciprocity in their organization (e.g., Batistič & Poell, 2022). To test this, we included reciprocity norms as a between-person variable (Eisenberger et al., 2004), including its interactions with daily knowledge sharing expectations, to determine whether the cross-level interaction – particularly between daily co-worker knowledge sharing expectations and learning goal orientation – remains significant when controlling for reciprocity norms.

## Study 2: method

### Transparency and openness

We described our sampling plan, all data exclusions, and all measures in the study. All data, analysis code, and research materials are available on the OSF page. This study’s design and analysis were preregistered (https://osf.io/m29tx).

### Procedure

The procedure of Study 2 was similar to Study 1. We used an ESM to collect daily diary data via Prolific Academic, employing the same sampling strategy as in Study 1. To ensure data quality, we included attention checks to verify whether participants followed instructions (Aguinis et al., 2013). The study was conducted between 12 October and 27 October, 2023. Participants were paid £1.35 for completing the baseline survey and received an additional £0.67 for each completed daily survey (duration: approximately 1 minute and 4 seconds). We measured the same variables as in Study 1 and additionally measured employees’ daily negative affect and daily general support in the daily surveys, and general organizational reciprocity norms in the baseline survey. The study received ethics approval (addendum #2022-WOP-15320).

### Sample

The initial sample consisted of 121 employed UK workers who filled out the baseline questionnaire, resulting in 935 daily observations. We removed one participant who did not complete the baseline questionnaire and five participants who did not complete any daily surveys. We included only daily observations where there was at least one interaction with either a supervisor or another co-worker, which led to the exclusion of 194 observations. We further excluded 14 daily observations where participants incorrectly answered the instrumental attention check. Finally, as in Study 1, we retained only employees who filled out at least two daily surveys, resulting in the exclusion of eight additional employees. Among the remaining 719 daily surveys, 500 represented days where an employee interacted only with their supervisor, 634 represented days with interactions only with a co-worker, and 415 represented days with interactions involving both their supervisor and a co-worker. Here we focused on the sample that included at least two daily surveys with interactions with both a supervisor and a co-worker, leading to the exclusion of an additional 14 participants. This resulted in a final sample of *N* = 401 days and *N* = 88 participants. On average, participants completed 4.56 daily surveys (*SD* = 2.37).

This final sample consisted of 41 men (46.60%) and 47 women (53.40%). The mean age of the participants was 39.00 years (*SD* = 9.56), and their mean organizational tenure at their current workplace was 7.41 years (*SD* = 6.78). Most employees worked between 31 and 40 hours per week (89.80%), while the remaining 10.20% worked between 41 and 50 hours per week. Moreover, 97.70% of participants held at least a high-school degree, and 84.10% had obtained a college or university degree. These demographics were similar to those in Study 1.

### Measures

All variables were assessed using a 5-point Likert-type response scale, ranging from 1 (*strongly disagree*) to 5 (*strongly agree*), unless otherwise stated. Table 3 presents the scales’ reliabilities, means, SDs, ICCs, and within-person and between-person correlations among the study variables.

**Table 3.** Descriptive statistics, Cronbach’s alphas, intraclass correlation coefficients, and correlations between study variables (study 2).

Variable		*M*	*SD*_b_	*SD*_w_	α_b_	α_w_	ICC	1	2	3	4	5	6	7	8	9
1	Daily supervisor knowledge sharing expectations	3.53	0.66	0.92	.90	.86	.31		.57**	.45**	−.33**	.75**	.49**	.36**	.30**	.16
2	Daily co-worker knowledge sharing expectations	3.74	0.58	0.78	.86	.81	.33	.41**		.72**	−.15	.44**	.63**	.26*	.33**	.12
3	Daily knowledge sharing	4.04	0.50	0.71	.93	.89	.37	.38**	.64**		−.16	.30*	.55**	.22*	.33**	.12
4	Daily negative affect	1.29	0.57	0.57	.95	.90	.78	−.20**	−.13*	−.14**		−.25*	−.10	−.05	.05	−.13
5	Daily general supervisor support	3.38	0.71	0.83	.88	.76	.50	.63**	.31**	.23**	−.17**		.59**	.19	.17	.15
6	Daily general co-worker support	3.49	0.59	0.73	.80	.72	.48	.31**	.55**	.47**	−.09	.42**		.12	.26*	.13
7	Learning goal orientation	3.82	0.79		.89										.47**	.09
8	Task interdependence	3.97	0.62		.81											.05
9	Reciprocity norms	3.36	0.59		.80											

Means, standard deviations at the between-person level (SD_b_) and within-person (i.e., day) level (SD_w_), and within-person correlations (*N* = 401; below diagonal) and between-person correlations (*N* = 88; above diagonal) correlations among study variables are displayed. Cronbach’s alphas for the between-person level (α_b_) and for the within-person level (α_w_) are depicted. ICC = intraclass correlation.

*p < .05; **p < .001.

#### Baseline survey measures

We used the same measures for learning goal orientation and task interdependence as in Study 1. Note that in Study 2, we included a reworded version of the negatively phrased item of the task interdependence scale that was excluded in Study 1 due to its impact on scale reliability (i.e., we removed “not” from the original item).^1^

#### Reciprocity norms in the organization

We measured stable reciprocity norms in the organization with the 8-item “positive reciprocity norm” scale developed by Eisenberger et al. (2004). We adjusted the original items from an interpersonal level to the organizational level. A sample item was: “If someone does me a favor in this organization, I feel obligated to repay them in some way”.

#### Daily survey measures

The day-specific measures for supervisor knowledge sharing expectations, co-worker knowledge sharing expectations, and knowledge sharing were identical to those used in Study 1.

##### Day-specific general supervisor [co-worker] support

We measured general supervisor [co-worker] support using a 4-item scale (Peeters & Le Blanc, 2001) that included items focusing on emotional support (“Today, my supervisor [co-workers] showed that they liked me”), appraisal support (“Today, my supervisor [co-workers] showed that they appreciate the way I do my work”), informational support (“Today, my supervisor [co-workers] gave me advice on how to handle things”), and instrumental support (“Today, my supervisor [co-workers] helped me with a given task”).

##### Day-specific negative affect

We measured negative affect using the short five-item version of the Positive and Negative Affect Schedule (PANAS; Watson et al., 1988). Participants rated how they felt for each of the following negative affective states on a given day on a scale ranging from 1 (*very slightly or not at all*) to 5 (*extremely*): distressed, upset, scared, jittery, and afraid.

### Measurement model

We evaluated the measurement model following the same procedure as in Study 1. First, we performed a MLCFA using Mplus 8 (Muthén & Muthén, 1998-2017). We compared a six-factor model (daily knowledge sharing, daily supervisor knowledge sharing expectations, daily co-worker knowledge sharing expectations, daily general supervisor support, daily general co-worker support and daily negative affect), specified on both the day level (within-level) and person level (between-level) simultaneously using MLR, with alternative models. The six-factor model showed a moderate fit (*χ*^2^(430) = 1053.07, *p* < .001, CFI = .848, TLI = .821, RMSEA = .060, SRMR_within_ = .058, SRMR_between_ = .113), and fit the data significantly better than any of the other nested models (see Table 4). Second, we conducted a CFA with the person-level variables. A three-factor model (learning goal orientation, task interdependence, and reciprocity norms) showed a moderate fit, *χ*^2^(132) = 235.65, *p* < .001, CFI = .839, TLI = .814, RMSEA = .044, SRMR = .096), and was significantly better than alternative two- and one-factor models (see Table 4).

**Table 4.** Results of the factor analyses for study 2.

Models	χ2	df	CFI	TLI	RMSEA	SRMR _within_	SRMR _between_
*Multilevel factor analysis with day-level variables*							
Six factor model	1053.07*	430	.848	.821	.060	.058	.113
Five factor model^a^	1116.53*	440	.835	.810	.062	.062	.128
Five factor model^b^	1107.28*	440	.837	.813	.061	.062	.124
Four factor model^c^	1158.79*	448	.827	.804	.063	.068	.143
Three factor model^d^	1788.19*	454	.675	.638	.086	.127	.151
Two-factor model^e^	2072.85*	458	.607	.565	.094	.123	.172
One factor model	2745.05*	460	.443	.388	.111	.148	.348
*Factor analysis with person-level variables*							
Three-factor model	235.65*	132	.839	.814	.044	–	.096
Two-factor model^f^	337.09*	134	.685	.640	.061	–	.118
One-factor model	504.61*fa	135	.426	.350	.083	–	.174

CFI = comparative fit index; TLI = Tucker – Lewis index; RMSEA = root mean square error of approximation; SRMR = standardized root mean square residual. ^a^Supervisor knowledge sharing expectations and general supervisor support as one factor. ^b^Co-worker knowledge sharing expectations and general co-worker support as one factor. ^c^Supervisor knowledge sharing expectations and general supervisor support as one factor and co-worker knowledge sharing expectations and general co-worker support as one factor. dSupervisor knowledge sharing expectations, general supervisor support, co-worker knowledge sharing expectations and general co-worker support as one factor. ^e^Supervisor knowledge sharing expectations, general supervisor support, co-worker knowledge sharing expectations, general co-worker support and knowledge sharing as one factory. ^f^Learning goal orientation and task interdependence as one factor.

*p < .001.

### Analytical strategy

We employed the same analytical strategy as in Study 1. Reciprocity norms in the organization was used as a Level 2 control variable and was grand-mean centred. Daily general supervisor and co-worker support were treated as Level 1 predictors and were person-mean centred. Additionally, daily negative affect, which was significantly correlated with daily knowledge sharing expectations and daily knowledge sharing, was included as a Level 1 control variable and was person-mean centred (Becker et al., 2016).

## Study 2: results

The output of the multilevel regression models are reported in Table 5. Results from Step 1 showed an ICC estimate of .37, indicating that 37% of the variance in knowledge sharing is between person and 63% is within person. In line with Hypothesis 1, daily perceived supervisor and co-worker knowledge sharing expectations were positively related to daily knowledge sharing (supervisor: estimate = 0.11, *SE* = 0.05, *p* = .030; co-worker: estimate = 0.36, *SE* = 0.09, *p* < .001). Daily general supervisor support was not significantly related to daily knowledge sharing (estimate = −0.07, *SE* = 0.05, *p* = .167). However, daily general co-worker support was significantly and positively related to daily knowledge sharing (estimate = 0.17, *SE* = 0.07, *p* = .014).

**Table 5.** Results of multilevel modelling analysis (study 2).

		*Model*					
		*Null (Step 1)*	*Random Intercept and Fixed Slope (Step 2)*	*Random Intercept and Random Slope (Step 3)*	*Cross-level interaction (Step 4a)*	*Cross-level interaction (Step 4b)*	*Cross-level interaction (Step 4)*
		*b (SE)*	*b* (SE)	*b* (SE)	*b* (SE)	*b* (SE)	*b* (SE)
*Fixed effects parameters*							
	*Within-person level*						
	Intercept	4.01*** (0.05)	4.06*** (0.06)	4.08** (0.03)	4.08*** (0.06)	4.08***(0.06)	4.08*** (0.35)
	Day		−0.01 (0.01)	−0.01 (0.01)	−0.01 (0.01)	−0.01 (0.01)	−0.01 (0.01)
	Daily negative affect		0.03 (0.07)	−0.02 (0.07)	−0.01 (0.07)	−0.02 (0.07)	−0.01 (0.07)
	Daily supervisor knowledge sharing expectations (DSKSE)		0.11* (0.05)	0.09* (0.04)	0.11* (0.04)	0.09** (0.04)	0.10* (0.04)
	Daily co-worker knowledge sharing expectations (DCKSE)		0.36*** (0.09)	0.41*** (0.08)	0.39*** (0.08)	0.42*** (0.08)	0.41*** (0.07)
	Daily general supervisor support		−0.07 (0.05)	−0.01 (0.04)	−0.02 (0.04)	−0.01 (0.04)	−0.01 (0.04)
	Daily general co-worker support		0.17* (0.07)	0.15* (0.06)	0.16 (0.06)	0.16 (0.06)	0.15* (0.06)
	*Between-person level*						
	Learning goal orientation (LGO)		0.06 (0.08)	0.04 (0.08)	0.05 (0.08)	0.04 (0.08)	0.06 (0.08)
	Task interdependence (TI)		0.21* (0.10)	0.25** (0.11)	0.24* (0.10)	0.23* (0.10)	0.22* (0.10)
	Reciprocity norms		0.08 (0.08)	0.10 (0.08)	0.10 (0.08)	0.10 (0.08)	0.10 (0.08)
	*Cross-level interactions*						
	DSKSE × LGO				0.05 (0.05)		0.02 (0.06)
	DCKSE × LGO				−0.17** (0.07)		−0.24** (0.07)
	DSKSE × TI					0.05 (0.05)	0.04 (0.06)
	DCKSE × TI					0.09 (0.12)	0.21 (0.12)
*Random effects parameters*							
	σ^2^ within	0.31*** (0.05)	0.22*** (0.03)	0.13*** (0.02)	0.13*** (0.02)	0.13*** (0.02)	0.13*** (0.02)
	σ^2^ between	0.18*** (0.04)	0.17*** (0.03)	0.19*** (0.03)	0.19*** (0.03)	0.19*** (0.03)	0.19*** (0.03)
	σ^2^ slope DSKSE			0.02 (0.03)	0.02 (0.02)	0.02 (0.02)	0.02 (0.02)
	σ^2^ slope DCKSE			0.19*** (0.05)	0.17** (0.05)	0.20*** (0.05)	0.17*** (0.05)
	ICC	0.37					
	Deviance (MLR)	385.54	324.19	292.15	289.47	291.11	287.30
	*R*^2^ (Level 1)		0.297	0.575	0.575	0.578	0.585
	*R*^2^ (Level 2)		0.051	−0.051	−0.056	−0.056	−0.056

MLR = maximum likelihood estimation with robust standard errors. Fixed effects represent the average relationships between the predictors and the outcomes across all individuals. Random effect parameters capture the variance in daily knowledge sharing at both the within- and between-person levels, as well as the variance in the slopes of daily knowledge-sharing expectations that is not explained by Level 2 predictors.

*p < .05. **p < .01. ***p < .001.

We also explored whether the strength of these estimates differed. Results showed that the difference in estimate strength between supervisor and co-worker knowledge sharing expectations was significant (difference = −0.26; *SE* = 0.10; *p* = .010), suggesting that co-worker knowledge sharing expectations had a stronger relationship with knowledge sharing compared to supervisor knowledge sharing expectations. The difference in estimate strength between supervisor knowledge sharing expectations and general co-worker support was not significant (difference = −0.06; *SE* = 0.09; *p* = .477). Similarly, the difference between co-worker knowledge sharing expectations and general co-worker support was also non-significant (difference = 0.19; *SE* = 0.13; *p* = .147).

**Figure 3.**
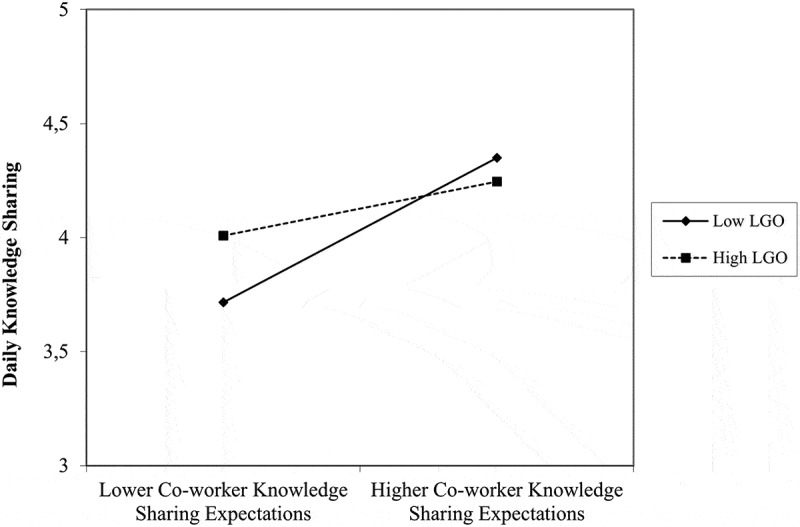
Dispositional learning goal orientation (LGO) as a cross-level moderator of the day-specific relationship between co-worker knowledge sharing expectations and knowledge sharing (study 2).

Learning goal orientation did not moderate the relationship between daily knowledge sharing expectations by supervisors and knowledge sharing (estimate = 0.02, *SE* = 0.06, *p* = .726). However, it did moderate the relationship between perceived daily co-worker knowledge sharing expectations and knowledge sharing (estimate = −0.24, *SE* = 0.07, *p* = .001). The interaction effect is visualized in Figure 3. Simple slope analysis showed that the positive relationship between daily perceived co-worker knowledge sharing expectations and daily knowledge sharing was stronger at lower levels of learning goal orientation (−1 SD: estimate = 0.60, *SE* = 0.10, *p* < .001) compared to higher levels of learning goal orientation (+1 SD: estimate = 0.22, *SE* = 0.09, *p* = .009). These finding supported Hypothesis 2, but for co-worker knowledge sharing expectations only. No significant interaction effect was found for task interdependence with daily supervisor knowledge sharing expectations (estimate = 0.04, *SE* = 0.06, *p* = .527) or co-worker knowledge sharing expectations (estimate = 0.21, *SE* = 0.12, *p* = .076). Thus, Hypothesis 3 was not supported.

To gain evidence for the robustness of our findings, additional analyses were conducted. First, we included cross-level interactions with reciprocity norms. These interactions were non-significant, indicating that the effect of co-worker knowledge sharing expectations was consistent regardless of the level of reciprocity norms. Additionally, the interaction between co-worker knowledge sharing expectations on knowledge sharing remained significant even when we controlled for reciprocity norms cross-level interactions. Furthermore, we reran the regression models, this time excluding daily negative affect, daily general supervisor and co-worker support, and reciprocity norms as covariates. The results remained similar when we excluded these covariates.

### Supplementary analyses

Supplementary analyses examined the relationships between daily supervisor and co-worker knowledge sharing expectations and knowledge sharing independently – without controlling for the other source of knowledge sharing expectations (see Tables S2.1 and S2.2 in the supplementary material). Results showed that the positive relationship between daily perceived supervisor knowledge sharing expectations and knowledge sharing remained significant (estimate = 0.20, *SE* = 0.07, *p* = .002; Table S2.1), as did the relationship for co-worker knowledge sharing expectations (estimate = 0.53, *SE* = 0.07, *p* < .001; Table S2.2). Furthermore, the interaction effect between co-worker knowledge sharing expectations and learning goal orientation remained significant (estimate = −0.21, *SE* = 0.06, *p* = .001; Table S2.2) and, in contrast to Study 1, was unaffected by the exclusion of supervisor knowledge sharing expectations as a covariate.

Finally, similar to Study 1, we examined whether knowledge sharing and knowledge sharing expectations on one day predicted knowledge sharing on the subsequent day (see Table S2.3). Knowledge sharing was negatively related to knowledge sharing the following day (*r* = −.18, *p* = .008). Neither supervisor daily knowledge sharing expectations (*r* = .07, *p* = .297) nor co-worker daily knowledge sharing expectations (*r* = −.09, *p* = .210) were significantly associated with next-day knowledge sharing.

## Discussion

Effectively managing knowledge poses a significant challenge for organizations. While previous research mainly focused on static relationships between general knowledge sharing and stable factors, more recent work shows that there is substantial variability in knowledge sharing from one day to the next (Li et al., 2022). To better understand the short-term nature of knowledge sharing and the factors that predict such behaviour within organizations, our study investigated the daily relationship between knowledge sharing expectations conveyed by supervisors and co-workers on the one hand, and employee knowledge sharing behaviours on the other hand.

The results of two preregistered studies, which employed a within-person diary design over 10 workdays, highlight that daily knowledge sharing expectations from supervisors and co-workers are positively associated with employees’ knowledge sharing on the same day. This relationship was consistently stronger when the expectations originated from co-workers. Neither study identified stable moderating effects of task interdependence. For learning goal orientation, we observed opposing moderating effects. In Study 1, the daily relationship was stronger for employees with high learning goal orientation, while in Study 2, it was stronger for those with low learning goal orientation. The latter finding was consistent with our hypothesis that high learning goal orientation may reduce the need for daily expectations to trigger knowledge sharing behaviours (i.e., substitute, cf. Kerr & Jermier, 1978). It is also important to note that the moderating role of learning goal orientation was specific to co-workers’ expectations and did not apply to supervisors’ expectations. Finally, supplementary analyses revealed that knowledge sharing on one day was negatively related to knowledge sharing the next day, while daily knowledge sharing expectations (from both supervisors and co-workers) did not predict next-day knowledge sharing behaviours.

### Theoretical implications

Our findings offer five important theoretical implications. First, we show that employees’ engagement in knowledge sharing varies daily, adding to the emerging understanding of knowledge sharing as a dynamic behaviour with episodic variance (Li et al., 2022). We identified daily supervisor and co-worker knowledge sharing expectations as important predictors of employees’ daily knowledge sharing behaviours. Specifically, our results indicate that these expectations are positively associated with knowledge sharing behaviour, beyond the associations observed with (general) daily work-based support and/or reciprocity norms within the organization. This suggests that employees not only share knowledge to reciprocate supportive behaviours (e.g., Cabrera et al., 2006; Halbesleben & Wheeler, 2015), but also that explicit expectations regarding the relevance and importance of knowledge sharing can further encourage this practice. The finding that such knowledge sharing expectations from the previous day were not related to next-day knowledge sharing further reinforces the importance of a within-level, day-to-day perspective: the effects of daily expectations may be somewhat fleeting, and expectations and other triggers for knowledge sharing may therefore need to be regularly reinforced.

Second, both studies found that co-worker expectations have a significantly stronger association with employee knowledge sharing than supervisor expectations, emphasizing the importance of peer dynamics. This suggests that co-workers play a more critical role in fostering knowledge sharing within organizations. Perhaps co-workers are more likely the targets of knowledge sharing, or they may interact more frequently with employees than supervisors do (Ferris & Mitchell, 1987). As such, co-workers may be more effective in encouraging knowledge sharing behaviours due to stronger social bonds and peer influence (Dannals et al., 2020; Siemsen et al., 2009). Alternatively, employees in similar roles might assume their knowledge is similar to that of their co-workers, reducing their intention to share it as they may not realize that their knowledge could help co-workers address daily challenges. This underscores the need for clear expectations and encouragement among co-workers to foster knowledge sharing (Israilidis et al., 2021). Overall, these findings imply that theories of knowledge sharing in organizations should place greater emphasis on horizontal (peer-to-peer) relationships rather than vertical (supervisor-subordinate) ones.

Third, our findings highlight the nuanced role of individual differences in motivational perspectives, particularly how learning goal orientation moderates responses to social expectations. The results of Study 1 showed that employees with a high learning goal orientation were more responsive to daily co-worker knowledge sharing expectations. The results of Study 2 showed the opposite: namely, that those with a low learning goal orientation were more responsive to daily co-worker knowledge sharing expectations. One explanation for Study 1’s findings is that employees with high learning goal orientation may view co-workers as allies in developing competence and mastering tasks, as knowledge sharing facilitates this process (Poortvliet et al., 2007). Another explanation could involve timing: Study 1 was conducted in summer 2022, during post-COVID-19 lockdowns and remote work policies, when co-worker interactions were limited, and role clarity was reduced. Employees with high learning goal orientation, naturally focused on improvement, may have been more sensitive to knowledge sharing cues in this context. By summer 2023, as COVID-related disruptions stabilized and the “new normal” took hold, employees with low learning goal orientation may have relied more on external triggers for knowledge sharing. These mixed results suggest that theories of self-regulation, particularly those addressing goal orientation (Janssen & Van Yperen, 2004; VandeWalle, 1997), should consider the interplay of personal and contextual factors to better understand how learning goal orientation predict daily knowledge sharing behaviour.

Fourth, the findings underscore the episodic and context-sensitive nature of knowledge sharing, as indicated by the lack of predictive power of task interdependence, and the negative relationship between knowledge sharing on consecutive days. Both studies found that higher knowledge sharing on one day corresponded to lower levels the next day, suggesting a reduced need to share knowledge or depleted resources. Similar patterns appear in other self-initiated and proactive behaviours: organizational citizenship behaviour has been linked to resource depletion (Koopman et al., 2016), taking charge has been linked to personal resource drain (Cangiano et al., 2021), and knowledge hiding has been linked to psychological strain responses (Venz & Nesher Shoshan, 2022) – all of which can hinder discretionary actions like knowledge sharing. Sijbom et al. (2024) similarly found that too many workplace changes decelerate learning, and suggested that employees may experience cognitive overload. Together, this challenges the notion that knowledge sharing is consistently sustained over time and highlights the role of temporal dynamics, such as resource depletion, fluctuating motivation, or perceived reciprocity. Theoretically, these results suggest that daily knowledge sharing is shaped more by situational and episodic factors than by stable traits or work-related conditions. Indeed, our findings reveal greater within-person variation than between-person variation in daily knowledge sharing. This reinforces the need to move beyond static models and incorporate dynamic, temporal perspectives to better capture the fluctuating nature of knowledge sharing behaviours.

Finally, neither study identified stable moderating effects of task interdependence. This finding aligns with the meta-analytic results of Kleingeld et al. (2011), who showed that task interdependence did not moderate the relationship between group goals (broadly aligned with expectations) and behavioural outcomes (e.g., group performance). One possible explanation is that task interdependence, while critical for coordinating actions among team members, may not influence knowledge sharing behaviours in response to expectations. Instead, in complex and ambiguous work environments, the focus may shift from task interdependence (the alignment of tasks and actions) to knowledge interdependence (the exchange of expertise and information). In these contexts, what employees know and share – rather than the tasks they perform – becomes central to performance. Given that knowledge interdependence often serves as a precursor to task interdependence in complex work settings (Raveendran et al., 2020), future research should investigate knowledge interdependence as a potential moderator in the relationship between daily knowledge-sharing expectations and actual knowledge-sharing behaviours.

### Practical implications

Our research holds several practical implications to foster knowledge sharing in organizations. First and foremost, our findings show that knowledge sharing is not a static behaviour but one that depends on daily triggers, such as expectations set by supervisors and co-workers. To capitalize on this, organizations should implement daily practices that make knowledge sharing an integral part of employees’ routines. In this, active and continued support is still needed, because knowledge sharing expectations are often not voiced (Israilidis et al., 2021) and may be somewhat fleeting. Supervisors can use morning briefings to outline specific knowledge-sharing needs for the day, and should clearly communicate the organization’s value and appreciation for knowledge sharing during regular workdays (Ellström & Ellström, 2014). They can also provide low-key daily feedback to stimulate knowledge sharing, which will build positive attitudes and experiences regarding these behaviours (cf. Hargadon & Bechky, 2006). By ensuring that knowledge sharing is continuously reinforced through explicit, daily cues, organizations can create a culture where sharing becomes habitual and naturally integrated into workflow processes.

Second, given the stronger association of co-worker knowledge sharing expectations compared to those of supervisors, organizations should focus on fostering peer collaboration to enhance knowledge sharing. Co-workers can collaborate to establish informal agreements or task-specific sharing expectations. This could involve creating team structures or peer-mentoring programmes that encourage open communication among employees. Tools like collaborative platforms for knowledge sharing might further strengthen these dynamics. Training managers to empower and facilitate peer interactions, rather than solely directing knowledge-sharing efforts themselves, can also be effective.

Third, organizations must recognize individual differences in employees’ responses to co-worker knowledge sharing expectations, as our results indicate varied reactions based on motivational orientations and/or contextual possibilities. For instance, employees with high learning goal orientation thrive with continuous learning opportunities but may need task-focused support to share knowledge in less interactive settings, akin to those with low learning goal orientation. Strategies like providing tools for peer collaboration – also in online or hybrid meetings–, mentoring opportunities, and recognition for knowledge sharing can help (Al-Alawi et al., 2007). Importantly, supervisors and co-workers should consistently express daily knowledge sharing expectations to all employees, regardless of their individual inclinations or contexts. In sum, embedding clear expectations for knowledge sharing into daily interactions – both from supervisors and co-workers – can transform it into a habit.

### Limitations and future research

While this study provides valuable insights into the dynamics of knowledge sharing, there are several limitations that should be acknowledged. First, while we measured our constructs on a daily basis, we assessed knowledge sharing expectations and knowledge sharing in a cross-sectional way at the end of the workday, reflecting how they relate to a particular day at work. Therefore, the causal relationships between daily knowledge sharing expectations and knowledge sharing cannot be established. Although we base ourselves on theories and earlier research that view the expression of expectations, whether related to knowledge or other desired work outcomes, as preceding work outcomes (Chiaburu & Harrison, 2008; Parker & Knight, 2024), the reverse might also be plausible.

Second, all variables were assessed via self-report measures, increasing the risk of mono-method bias (Podsakoff et al., 2012). We believe self-reports were appropriate because employees generally have the most accurate insight into their own knowledge sharing and subjective perceptions of knowledge sharing expectations. However, to strengthen validity and reliability of the data, we implemented several precautions. For example, we controlled for daily negative affect to mitigate self-rating concerns (Gabriel et al., 2019) and measured between-person variables separately from day-specific variables. Nevertheless, the measurement of knowledge sharing may still be susceptible to social desirability bias, potentially leading to overreporting. While we included daily negative affect as a control variable, future research should consider measuring negative affect separately from the focal variables to better understand its directionality in relation to knowledge sharing. Additionally, incorporating positive affect would offer a more comprehensive view of the factors that play a role in daily knowledge sharing (Gabriel et al., 2019).

Third, the specific mechanisms underlying the relationships between knowledge sharing expectations and knowledge sharing behaviour remain unclear. Although our findings suggest the applicability of role perception theory (Dierdorff & Morgeson, 2007) and a cost-benefit framework (cf. Morrison & Vancouver, 2000; Wang & Noe, 2010), it is uncertain whether increased clarity regarding knowledge sharing in one’s work role and decreased risks and/or increased benefits indeed serve as mediating mechanisms. Future research could explore these underlying principles by measuring them directly. It would be particularly valuable to examine whether these mechanisms differ across sources of knowledge sharing expectations, as our findings indicate that co-worker expectations more strongly predict knowledge sharing behaviour than those from supervisors.

Fourth, the fit of the measurement model in Study 2 was suboptimal suggesting a discrepancy between the model-derived covariance matrix and the data-informed covariance matrix (Bazzoli, 2024). To further examine the robustness of our findings, we reran the MLCFA of Study 2 focusing only on our focal variables, and this model had a better fit (χ^2^(64) = 82.626, *p* = .058, CFI = .988, TLI = .984, RMSEA = .027, SRMR_within_ = .029, SRMR_between_ = .061). This could mean that the poorer fit could be due to the inclusion of daily general supervisor and co-worker support, which are strongly correlated with daily supervisor and co-worker knowledge sharing expectations.

Finally, the diverse organizational backgrounds of our research samples enhance the generalizability of our findings. However, as both samples come from the UK, with a predominantly individualistic culture, the applicability to other cultural contexts may be limited. In collectivistic cultures, the link between knowledge sharing expectations and behaviour may be stronger than observed here. For example, Chow et al. (2000) found that Chinese individuals exhibited greater willingness to share knowledge, prioritizing collective interests over personal gain, particularly when the knowledge can benefit the organization despite harming the sharer’s self-interest.

## Conclusion

This study underscores the significance of understanding the dynamics of daily knowledge sharing in the workplace. Our findings highlight the critical and on-going role of communicated expectations from supervisors and – especially – from co-workers in predicting employees’ knowledge-sharing behaviours. The link between co-worker knowledge sharing expectations and knowledge sharing depended on employees’ learning goal orientation – albeit with mixed patterns across studies. These findings add value by demonstrating that knowledge sharing is not only predicted by structural and organizational factors but also by nuanced, daily interactions between individuals at work, thereby advancing our understanding of how to foster knowledge sharing in organizational settings.

## Supplementary Material

Table S1_3 Supplementary correlations with lagged results_Study1.docx

Table S1_2 Supplementary multilevel analyses results coworkerrs only_Study1.docx

Table S2_2 Supplementary multilevel analyses results coworkerrs only_Study 2.docx

Table S2_1 Supplementary multilevel analyses results supervisors only_Study2.docx

Table S2_3 Supplementary correlations with lagged results_Study2.docx

Table S1_1 Supplementary multilevel analyses results supervisors only_Study1.docx

## Data Availability

The data and syntax that support the findings of this study are openly available in OSF at https://osf.io/9tqj4/?view_only=cbdde830ee0e465887a5902fbe793e61.
