# Supplementary material for: Daily knowledge sharing at work: the role of daily knowledge sharing expectations, learning goal orientation and task interdependence
Source: Eur J Work Organ Psychol. 2025 Jan 30;34(2):298–314. doi: 10.1080/1359432X.2025.2458343 (PMC11934953; doi:10.1080/1359432X.2025.2458343)
Supplement: Table S1_3 Supplementary correlations with lagged results_Study1.docx [file PEWO_A_2458343_SM9068.docx]

| Table S1.3 | |  |  |  |  |  |  |  |
| --- | --- | --- | --- | --- | --- | --- | --- | --- |
| *Descriptive statistics, Cronbach's alphas, intraclass correlation coefficients, and correlations between study variables at the within-person level.* | | | | | | | | |
| **Variable** | | ***M*** | ***SD*** | **1** | **2** | **3** | **4** | **5** |
| 1 | Daily supervisor knowledge sharing expectations | 3.88 | 0.82 |  |  |  |  |  |
| 2 | Daily co-worker knowledge sharing expectations | 3.95 | 0.70 | .48** |  |  |  |  |
| 3 | Daily knowledge sharing | 4.16 | 0.65 | .44** | .71** |  |  |  |
| 4 | Lagged daily supervisor knowledge sharing expectations^a^ | -0.02 | 0.60 | -.11* | .05 | -.03 |  |  |
| 5 | Lagged daily co-worker knowledge sharing expectations^a^ | 0.00 | 0.47 | -.09 | -.11 | -.10 | .31** |  |
| 6 | Lagged daily knowledge sharing^a^ | 0.02 | 0.49 | -.08 | -.08 | -.17* | .37** | .64** |
|  |  |  |  |  |  |  |  |  |
| Note: Means, standard deviations at the within-person (i.e., day) level (SD), and within-person correlations (*N* = 577). | | | | | | | | |
| * *p* < .05; ** *p* < .001. | |  |  |  |  |  |  |  |
| ^a^ *N* = 332 | |  |  |  |  |  |  |  |
