# Supplementary material for: Daily knowledge sharing at work: the role of daily knowledge sharing expectations, learning goal orientation and task interdependence
Source: Eur J Work Organ Psychol. 2025 Jan 30;34(2):298–314. doi: 10.1080/1359432X.2025.2458343 (PMC11934953; doi:10.1080/1359432X.2025.2458343)
Supplement: Table S1_2 Supplementary multilevel analyses results coworkerrs only_Study1.docx [file PEWO_A_2458343_SM9067.docx]

| Table S1.2 | |  |  |  |  |
| --- | --- | --- | --- | --- | --- |
| Supplementary analyses with data from co-workers only (Study 1) | | | | |  |
|  |  | ***Model*** | | | |
|  |  | ***Null (Step 1)*** | ***Random Intercept and Fixed Slope (Step 2)*** | ***Random Intercept and Random Slope (Step 3)*** | ***Cross-level interaction (Step 4)*** |
|  |  | *b (SE)* | *b* (SE) | *b* (SE) | *b* (SE) |
| *Fixed effects parameters* | |  |  |  |  |
|  | *Within-person level* |  |  |  |  |
|  | Intercept | 4.10***(0.04) | 4.12^***^ (0.05) | 4.12^***^ (0.05) | 4.12^***^ (0.05) |
|  | Day |  | -0.00 (0.01) | -0.00 (0.01) | -0.00 (0.01) |
|  | Daily co-worker knowledge sharing expectations (DCKSE) |  | 0.67^***^ (0.05) | 0.59^***^ (0.05) | 0.59^***^ (0.04) |
|  |  |  |  |  |  |
|  | *Between-person level* |  |  |  |  |
|  | Learning goal orientation (LGO) |  | 0.24^***^ (0.06) | 0.24*** (0.06) | 0.23^***^ (0.06) |
|  | Task interdependence (TI) |  | 0.17* (0.08) | 0.20^**^ (0.07) | 0.18* (0.08) |
|  |  |  |  |  |  |
|  | *Cross-level interactions* |  |  |  |  |
|  | DCKSE × LGO |  |  |  | 0.03 (0.08) |
|  | DCKSE × TI |  |  |  | 0.07 (0.10) |
|  |  |  |  |  |  |
| *Random effects parameters* | |  |  |  |  |
|  | σ^2^ within | 0.35*** (0.04) | 0.20*** (0.02) | 0.17*** (0.02) | 0.17*** (0.02) |
|  | σ^2^ between | 0.15*** (0.02) | 0.14*** (0.02) | 0.15*** (0.02) | 0.15*** (0.02) |
|  | σ^2^slope DCKSE |  |  | 0.09*** (0.03) | 0.09** (0.03) |
|  |  |  |  |  |  |
|  | Deviance (MLR) | 738.09 | 549.83 | 524.57 | 524.09 |
|  |  |  |  |  |  |
| Note: MLR = maximum likelihood estimation with robust standard errors. Fixed effects represent the average relationships between the predictors and the outcomes across all individuals. Random effect parameters capture the variance in daily knowledge sharing at both the within- and between-person levels, as well as the variance in the slopes of daily knowledge-sharing expectations that is not explained by Level 2 predictors. | | | | | |
| * *p* < .05. ** *p* < .01. *** *p* < .001. | | |  |  |  |
