# Supplementary material for: Daily knowledge sharing at work: the role of daily knowledge sharing expectations, learning goal orientation and task interdependence
Source: Eur J Work Organ Psychol. 2025 Jan 30;34(2):298–314. doi: 10.1080/1359432X.2025.2458343 (PMC11934953; doi:10.1080/1359432X.2025.2458343)
Supplement: Table S2_1 Supplementary multilevel analyses results supervisors only_Study2.docx [file PEWO_A_2458343_SM9065.docx]

| Table S2.1 | |  |  |  |  |
| --- | --- | --- | --- | --- | --- |
| Supplementary analyses with data from supervisors only (Study 2) | | | | |  |
|  |  | ***Model*** | | | |
|  |  | ***Null*** | ***Random Intercept and Fixed Slope*** | ***Random Intercept and Random Slope*** | ***Cross-level interaction*** |
|  |  | *b (SE)* | *b* (SE) | *b* (SE) | *b* (SE) |
| *Fixed effects parameters* | |  |  |  |  |
|  | *Within-person level* |  |  |  |  |
|  | Intercept | 3.77*** (0.06) | 3.86*** (0.08) | 3.85** (0.08) | 3.86^***^ (0.08) |
|  | Day |  | -0.02 (0.01) | -0.01 (0.01) | -0.02 (0.01) |
|  | Daily negative affect |  | 0.10 (0.09) | 0.09 (0.09) | 0.09 (0.09) |
|  | Daily supervisor knowledge sharing expectations (DSKSE) |  | 0.20^**^ (0.07) | 0.21** (0.06) | 0.22^**^ (0.07) |
|  | Daily general supervisor support |  | 0.04 (0.08) | 0.02 (0.07) | 0.01 (0.08) |
|  |  |  |  |  |  |
|  | *Between-person level* |  |  |  |  |
|  | Learning goal orientation (LGO) |  | 0.03 (0.09) | 0.06 (0.09) | 0.03 (0.09) |
|  | Task interdependence (TI) |  | 0.21* (0.10) | 0.20*^*^ (0.10) | 0.21* (0.10) |
|  | Reciprocity norms |  | 0.12 (0.08) | 0.09 (0.08) | 0.09 (0.08) |
|  |  |  |  |  |  |
|  | *Cross-level interactions* |  |  |  |  |
|  | DSKSE × LGO |  |  |  | 0.14 (0.07) |
|  | DSKSE × TI |  |  |  | -0.10 (0.13) |
|  |  |  |  |  |  |
| *Random effects parameters* | |  |  |  |  |
|  | σ^2^ within | 0.58*** (0.07) | 0.55*** (0.06) | 0.52*** (0.06) | 0.52*** (0.07) |
|  | σ^2^ between | 0.24*** (0.05) | 0.22*** (0.04) | 0.23*** (0.04) | 0.23*** (0.04) |
|  | σ^2^slope DSKSE |  |  | 0.05 (0.04) | 0.04 (0.04) |
|  |  |  |  |  |  |
|  | Deviance (MLR) | 616.77 | 602.36 | 599.67 | 598.18 |
|  |  |  |  |  |  |
|  |  |  |  |  |  |
| Note: MLR = maximum likelihood estimation with robust standard errors. Fixed effects represent the average relationships between the predictors and the outcomes across all individuals. Random effect parameters capture the variance in daily knowledge sharing at both the within- and between-person levels, as well as the variance in the slopes of daily knowledge-sharing expectations that is not explained by Level 2 predictors. | | | | | |
| * *p* < .05. ** *p* < .01. *** *p* < .001. | | |  |  |  |
