# Supplementary material for: Daily knowledge sharing at work: the role of daily knowledge sharing expectations, learning goal orientation and task interdependence
Source: Eur J Work Organ Psychol. 2025 Jan 30;34(2):298–314. doi: 10.1080/1359432X.2025.2458343 (PMC11934953; doi:10.1080/1359432X.2025.2458343)
Supplement: Table S2_3 Supplementary correlations with lagged results_Study2.docx [file PEWO_A_2458343_SM9064.docx]

| Table S2.3 | |  |  |  |  |  |  |  |  |  |
| --- | --- | --- | --- | --- | --- | --- | --- | --- | --- | --- |
| *Descriptive statistics, Cronbach's alphas, intraclass correlation coefficients, and correlations between study variables at the within-person level.* | | | | | | | | | | |
| **Variable** | | ***M*** | ***SD*** | **1** | **2** | **3** | **4** | **5** | **6** | **7** |
| 1 | Daily supervisor knowledge sharing expectations | 3.53 | 0.92 |  |  |  |  |  |  |  |
| 2 | Daily co-worker knowledge sharing expectations | 3.74 | 0.78 | .41** |  |  |  |  |  |  |
| 3 | Daily knowledge sharing | 4.04 | 0.71 | .38** | .64** |  |  |  |  |  |
| 4 | Daily negative affect | 1.29 | 0.57 | -.20** | -.13* | -.14* |  |  |  |  |
| 5 | Lagged daily supervisor knowledge sharing expectations^a^ | -0.02 | 0.69 | -.13 | -.10 | -.05 | .04 |  |  |  |
| 6 | Lagged daily co-worker knowledge sharing expectations^a^ | -0.04 | 0.53 | .05 | -.17* | -.01 | -.10 | .30** |  |  |
| 7 | Lagged daily knowledge sharing^a^ | -0.01 | 0.46 | .07 | -.09 | -.18* | -.00 | .25** | .55** |  |
| 8 | Lagged daily negative affect^a^ | 0.00 | 0.23 | -.07 | -.05 | -.00 | .06 | .03 | -.03 | -.05 |
| Note: Means, standard deviations at the within-person (i.e., day) level (SD), and within-person correlations (*N* = 401). | | | | | | | | |  |  |
| * *p* < .05; ** *p* < .001. | |  |  |  |  |  |  |  |  |  |
| ^a^ *N* = 213 | |  |  |  |  |  |  |  |  |  |
